# Reporting Tumor Molecular Heterogeneity in Histopathological Diagnosis

**DOI:** 10.1371/journal.pone.0104979

**Published:** 2014-08-15

**Authors:** Andrea Mafficini, Eliana Amato, Matteo Fassan, Michele Simbolo, Davide Antonello, Caterina Vicentini, Maria Scardoni, Samantha Bersani, Marisa Gottardi, Borislav Rusev, Giorgio Malpeli, Vincenzo Corbo, Stefano Barbi, Katarzyna O. Sikora, Rita T. Lawlor, Giampaolo Tortora, Aldo Scarpa

**Affiliations:** 1 Applied Research on Cancer Network (ARC-NET) and Department of Pathology and Diagnostics, University and Hospital Trust of Verona, Verona, Italy; 2 Department of Surgery, University and Hospital Trust of Verona, Verona, Italy; 3 Department of Medicine, Oncology Unit, University and Hospital Trust of Verona, Verona, Italy; National Cancer Institute, National Institutes of Health, United States of America

## Abstract

**Background:**

Detection of molecular tumor heterogeneity has become of paramount importance with the advent of targeted therapies. Analysis for detection should be comprehensive, timely and based on routinely available tumor samples.

**Aim:**

To evaluate the diagnostic potential of targeted multigene next-generation sequencing (TM-NGS) in characterizing gastrointestinal cancer molecular heterogeneity.

**Methods:**

35 gastrointestinal tract tumors, five of each intestinal type gastric carcinomas, pancreatic ductal adenocarcinomas, pancreatic intraductal papillary mucinous neoplasms, ampulla of Vater carcinomas, hepatocellular carcinomas, cholangiocarcinomas, pancreatic solid pseudopapillary tumors were assessed for mutations in 46 cancer-associated genes, using Ion Torrent semiconductor-based TM-NGS. One ampulla of Vater carcinoma cell line and one hepatic carcinosarcoma served to assess assay sensitivity. *TP53*, *PIK3CA*, *KRAS*, and *BRAF* mutations were validated by conventional Sanger sequencing.

**Results:**

TM-NGS yielded overlapping results on matched fresh-frozen and formalin-fixed paraffin-embedded (FFPE) tissues, with a mutation detection limit of 1% for fresh-frozen high molecular weight DNA and 2% for FFPE partially degraded DNA. At least one somatic mutation was observed in all tumors tested; multiple alterations were detected in 20/35 (57%) tumors. Seven cancers displayed significant differences in allelic frequencies for distinct mutations, indicating the presence of intratumor molecular heterogeneity; this was confirmed on selected samples by immunohistochemistry of p53 and Smad4, showing concordance with mutational analysis.

**Conclusions:**

TM-NGS is able to detect and quantitate multiple gene alterations from limited amounts of DNA, moving one step closer to a next-generation histopathologic diagnosis that integrates morphologic, immunophenotypic, and multigene mutational analysis on routinely processed tissues, essential for personalized cancer therapy.

## Introduction

Cancer inter-tumor and intra-tumor heterogeneity, a well-known fact described by pathologists in the classification of tumors over the last two centuries, has finally risen to the forefront of clinical interest. Cancer genomics and transcriptomics studies have shown that tumors belonging to the same histotype display remarkable differences in their genetic assets; such inter-tumor heterogeneity is the basis of molecular subclassification with clinical impact for targeted therapeutic approaches. It has also become clear that phenotypically and genetically diverse clones of neoplastic cells may be juxtaposed within the same tumor[1], [2]. These clones are thought to be players in a branching clonal evolution scenario leading to the formation of metastases that are more aggressive and resistant to treatments than the primary tumor [1].

The histological and immunohistochemical characterization of multiple samples from the same tumor can highlight the presence of subpopulations of neoplastic cells displaying peculiar morphological and immunophenotypical features; this morpho-phenotypical analysis of intratumor heterogeneity finds its natural complement in a comprehensive characterization of molecular lesions within a cancer specimen. The sum of these data offers essential information to diagnose and subclassify cancers for the scope of determining prognosis and selecting tailored treatments [3].

The sequencing analysis of hotspot mutations in cancer-related genes has thus become a useful tool in selecting personalized therapy for many malignancies [4], [5]. However, the use of conventional techniques for a wide molecular characterization of tumors is hampered by the high costs and time needed to assess multiple molecular alterations, and by the limited amount of tissue consisting in formalin-fixed paraffin-embedded (FFPE) biopsies and/or fine needle aspiration cytology. This calls for the implementation of companion diagnostic methods for (i) simultaneously testing multiple genetic alterations and (ii) quantifying the molecular subclones, i.e. the amount of cancer cells harboring any different mutation.

Massive parallel sequencing, also known as next-generation sequencing (NGS), has recently been introduced and is the most sensitive approach to index multiple genes starting from a limited amount of DNA [6], [7]. In the present study, we assayed a targeted multigene NGS (TM-NGS) test in 35 FFPE samples from diverse upper gastrointestinal tract tumors to define its diagnostic potential in characterizing cancer molecular heterogeneity.

## Materials and Methods

### Tumor samples

A series of 35 formalin-fixed paraffin-embedded (FFPE) samples from surgically resected neoplasms, representative of diverse upper gastrointestinal and hepatobiliopancreatic cancer types (**Table S1**), were assayed for intragenic mutations in 46 cancer-related genes by TM-NGS. The series included 5 intestinal type gastric carcinomas (GC), 5 pancreatic ductal adenocarcinomas (PDAC), 5 pancreatic intraductal papillary mucinous neoplasms (IPMN), 5 ampulla of Vater carcinomas (AVC), 5 hepatocellular carcinomas (HCC), 5 intrahepatic cholangiocarcinomas (ICC), and 5 pancreatic solid pseudopapillary tumors (SPT). These latter had matched fresh-frozen and FFPE samples available and served to assess the performance of TM-NGS on DNA from both sources. In addition, DNA from cancer cell line AVC1 [8] and one hepatic carcinosarcoma [9], served to assess sensitivity of the TM-NGS mutational assay.

### Ethics

A total of 35 samples from 35 patients, acquired by the ARC-Net biobank at the University and Hospital Trust of Verona - Italy, were used in the present study. Of these, the materials from 9 patients have been collected with the written informed consent for their use in research under Program 1885 (creation of a biobank) protocol 52438 approved by the local ethics committee of the Integrated Unversity Hospital Trust of Verona on November 23rd 2010. This approval covers biological material collection for the ARC-Net coordinated biobank of samples from all cancer patients, including neoplastic and associated local and distant normal tissue. Protocols for collection included informed consent, approved under this program, from the patient to collect residual tissue samples for molecular research. The program includes approved amendments to address the later regulatory issues of sensitive data in genomic studies and a separate informed consent for access to sensitive data. These informed consents, received from patients, are registered in the biobank database together with samples collected. Samples from the remaining 26 patients had been collected prior to November 23rd 2010. These samples were acquired by the biobank according to a protocol approved by the local ethics committee for use in research of residual pathological tissue under an amendment to the above mentioned program 1885 (creation of a biobank). The protocol indicates the procedure to acquire and register these samples, and anonymize patient information where it was not reasonable to reconsent patients directly. Thus, the consent for use of samples from these 26 patients was waived by the ethics commitee.

### DNA extraction and qualification

Neoplastic cellularity was assessed by microscopic examination and, when below 50%, enriched by manually microdissecting four consecutive 10 µm thick sections. All samples were microdissected excluding the five SPT, HCC1 to HCC4, and ICC1.

Genomic DNA from frozen or FFPE tissues was extracted using the QiAamp DNA Mini Kit or QIAamp DNA FFPE Tissue Kit (Qiagen), respectively. Purified DNA was quantified and its quality assessed using Qubit (Invitrogen Life Technologies) and NanoDrop (Invitrogen Life Technologies) platforms [10]. DNA suitability for PCR downstream applications was further evaluated through BIOMED 2 PCR multiplex protocol and the PCR products were evaluated by DNA 1000 Assay (Invitrogen Life Technologies) on the Agilent 2100 Bioanalyzer on-chip electrophoresis (Agilent Technologies) [11].

### Targeted Multigene Next Generation Sequencing of Multiplex PCR Amplicons

Twenty ng of DNA were used for multiplex PCR amplification using the Ion AmpliSeq Cancer Panel (Life Technologies) that explores selected regions of the following 46 cancer-associated genes: *ABL1, ALK, AKT1, APC, ATM, BRAF, CDH1, CDKN2A, CSF1R, CTNNB1, EGFR, ERBB2, ERBB4, FBXW7, FGFR1, FGFR2, FGFR3, FLT3, GNAS, HNF1A, HRAS, IDH1, JAK2, JAK3, KDR/VEGFR2, KIT, KRAS, MET, MLH1, MPL, NOTCH1, NPM1, NRAS, PDGFRA, PIK3CA, PTEN, PTPN11, RET, RB1, SMAD4, SMARCB1, SMO, SRC, STK11, TP53, VHL*.

The quality of the obtained library was evaluated by the Agilent 2100 Bioanalyzer on-chip electrophoresis (Agilent Technologies). Emulsion PCR was performed either manually or with the OneTouch DL system (Life Technologies). Sequencing was run on the Ion Torrent Personal Genome Machine (PGM, Life Technologies) loaded with a 316 chip as per manufacturer's protocol. Data analysis, including alignment to the hg19 human reference genome as well as variant calling and filtering, was done using the Torrent Suite Software v.3.6 (Life Technologies) with default options for the Ion AmpliSeq Cancer Panel. Variants were annotated using the SnpEff software v.3.4 [12] and the NCBI mRNA Reference Sequences listed in **Table S2**. Alignments were visually verified with the Integrative Genomics Viewer; IGV v.2.3 [13].

### DNA Sanger Sequencing and Immunohistochemistry

To validate the mutations detected by TM-NGS, *TP53* (exons 5, 6, 7, 8), *PIK3CA* (exon 10), *KRAS* (exon 2) and *BRAF* (exon11 and exon15) specific PCR fragments were analyzed by conventional Sanger sequencing, as described previously [14]–[16]. The immunohistochemical expression of p53 (clone DO-1, Immunotech, dilution 1∶50) and Smad4 (clone B8, Santa Cruz Biotechnology, dilution 1∶200) was tested as a surrogate validation of the TM-NGS results for these genes. The protocol included deparaffination of 4- µm FFPE sections in xylene, rehydration via decreasing concentrations of alcohol down to pure water, non-enzymatic antigen retrieval in citrate buffer (pH 6.0) for 30 minutes at 95°C. Immunolabeling was developed using the Novolink polymer detection kit (Leica Microsystems) according to the manufacturer instructions. Appropriate positive and negative controls were run concurrently.

## Results

### TM-NGS yields overlapping results on DNA from frozen and paraffin samples and quantitates the mutated alleles

**Figure 1:**
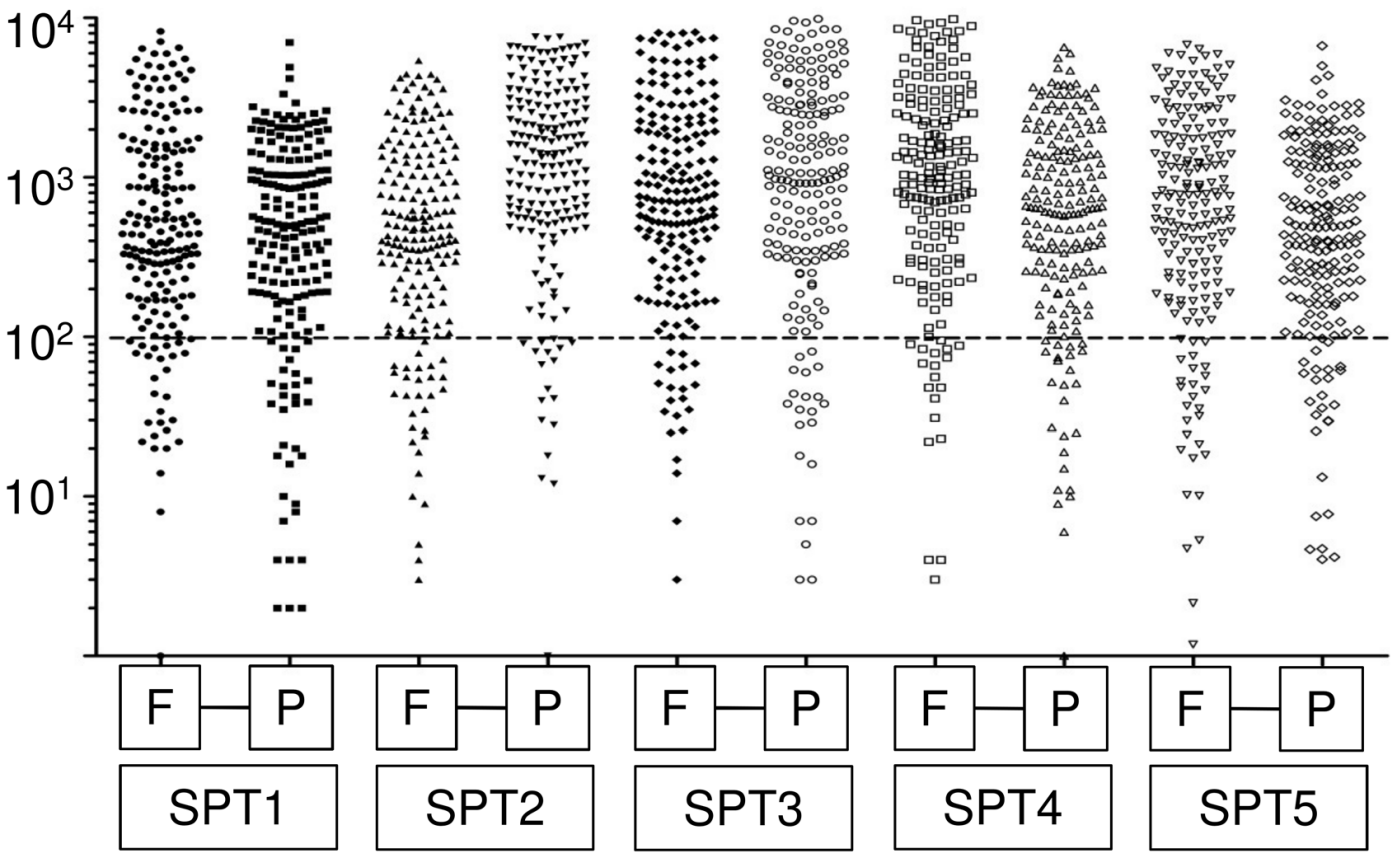
Targeted multigene-next generation sequencing analysis of five solid pseudopapillary tumors. Depth of sequencing (coverage) of targeted regions analyzed in 5 matched fresh-frozen and formalin-fixed paraffin embedded samples of solid pseudopapillary tumor. Dots describe the coverage of each target sequence per sample; the quantity of sequences obtained was similar for fresh-frozen (F) and formalin-fixed paraffin embedded (P) derived DNA.

We used 5 SPT for which matched fresh-frozen and FFPE samples were available to test the proficiency of the assay using DNA from FFPE tissues. This tumor type is ideal for this purpose because it is characterized by a monotonous composition with a neoplastic cellularity of about 70–80% and has a molecular hallmark consisting of a heterozygous *CTNNB1* mutation [17]. Both fresh-frozen and FFPE specimens were assessed for neoplastic cellularity by two independent pathologists and the extracted DNA was subjected to TM-NGS. The quantity of sequences obtained was similar for fresh-frozen and FFPE derived DNA (**Figure 1**). The *CTNNB1* gene mutation was detected in all samples; moreover, the allelic frequency of *CTNNB1* mutation was consistent with the percentage of tumor cells as scored by the pathologists (**Table 1**, figure 1).

**Table 1:** Concordance between tumor cellularity and *CTNNB1*/β-catenin mutation prevalence detected by deep sequencing in five solid pseudopapillary tumors (SPT).

Case ID	Sample type	Tumor cells	*CTNNB1* mutation allelic frequency
SPT1	Frozen	80%	39%
SPT1	FFPE*	80%	39%
SPT2	Frozen	80%	42%
SPT2	FFPE	80%	42%
SPT3	Frozen	80%	46%
SPT3	Frozen	80%	39%
SPT4	Frozen	70%	27%
SPT4	Frozen	70%	42%
SPT5	Frozen	70%	28%
SPT5	FFPE	70%	36%

*FFPE  =  formalin-fixed, paraffin embedded specimen.

### TM-NGS is highly sensitive on DNA from both frozen and paraffin tissue

**Figure 2:**
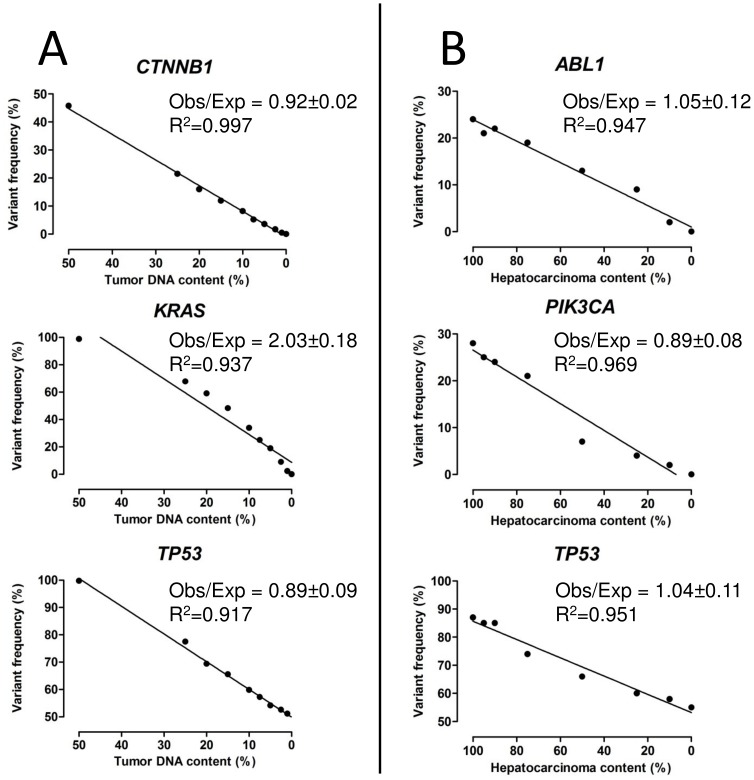
Sensitivity of TM-NGS for mutation assessment in fresh-frozen and formalin-fixed paraffin embedded samples. **A**) DNA from AVC1 cell line and a commercial germline DNA were mixed to obtain samples with a decreasing relative AVC1 DNA content (50%, 25%, 20%, 15%, 10%, 7.5%, 5%, 2.5%, 1% and 0%) to test TM-NGS sensitivity on DNA from frozen tissues. Three known homozygous variants harbored by the AVC1 cell line (*KRAS* G12A, *CTNNB1* S45F and the nonpathogenic polymorphism *TP53* P72R) were used to assess the assay sensitivity. The commercial germline DNA was heterozygous for the *TP53* P72R nonpathogenic polymorphism. The variants were identified in all samples containing AVC1 DNA, down to a frequency of 1%. Obs: mutation frequency detected by instrument, Exp: expected value calculated basing on dilution and mutation allelic frequency in the source AVC1 DNA. B) A case of carcinosarcoma was used to test TM-NGS sensitivity in formalin-fixed paraffin embedded samples. DNA from two separate tumor components (hepatocarcinoma and sarcoma) was mixed to obtain samples with a decreasing relative hepatocarcinoma DNA content: 100%, 95%, 90%, 75%, 50%, 25%, 10% and 0%. These were subjected to the assay exploiting three known different mutations: *ABL1* intronic g.164164 G>T (variant frequency hepatocarcinoma = 24% sarcoma = 0%), *PIK3CA* H1047R (variant frequency hepatocarcinoma = 28% sarcoma = 0%) and *TP53* F109C (variant frequency hepatocarcinoma = 87%; sarcoma = 55%) The three mutations were identified in all samples containing hepatocarcinoma DNA down to a frequency of 2%, corresponding to the frequency of *ABL1* gene mutation in the 10% diluted sample. Obs: mutation frequency detected by instrument, Exp: expected value calculated basing on hepatocarcinoma/sarcoma mixing ratio and mutation allelic frequency in each tumor component before dilution.

The limit of mutation detection of TM-NGS on DNA from fresh-frozen samples was assessed using DNA from AVC1 cancer cell line with known mutations [8] serially diluted with non-tumor DNA from a commercial source (Universal unmethylated DNA, Chemicon Int., Billerica, MA). AVC1 cell line harbors the following homozygous variants: *KRAS* G12A and *CTNNB1* S45F somatic mutations and the nonpathogenic *TP53* P72R variant. The commercial DNA is heterozygous for the common *TP53* P72R nonpathogenic polymorphism. AVC1 and commercial DNA were mixed to obtain samples with a decreasing relative AVC1 DNA content: 50%, 25%, 20%, 15%, 10%, 7.5%, 5%, 2.5%, 1% and 0%. Twenty ng of each dilution point were subjected to TM-NGS with the Ion AmpliSeq Cancer Panel. The three variants were identified in all samples containing AVC1 DNA down to a frequency of 1% (**Figure 2A**). The ratio between tumor DNA content and allelic frequency was approaching one for *CTNNB1* and *TP53* variants, while *KRAS* mutation showed a higher ratio (2.03±0.18). This is consistent with the previous AVC1 characterization showing copy number gain of chromosome 12p, where *KRAS* resides [8].

To assess whether TM-NGS has the same detection limit on DNA from FFPE, we used two different tumor components from a previously characterized hepatic carcinosarcoma [9]. DNA from the microdissected hepatocarcinoma and sarcoma components was mixed to obtain samples with a decreasing relative hepatocarcinoma DNA content: 100%, 95%, 90%, 75%, 50%, 25%, 10% and 0% (**Figure 2B**). Twenty ng of each dilution point were subjected to TM-NGS with the Ion AmpliSeq Cancer Panel. Three known different genetic variants of the hepatocarcinoma component were used to assess the assay sensitivity: *ABL1* intronic g.164164 G>T (variant frequency hepatocarcinoma = 24% sarcoma = 0%), *PIK3CA* H1047R (variant frequency hepatocarcinoma = 28% sarcoma = 0%) and *TP53* F109C (variant frequency hepatocarcinoma = 87%; sarcoma = 55%). The three mutations were identified in all samples containing hepatocarcinoma DNA down to a frequency of 2%, corresponding to the frequency of *ABL1* gene mutation in the 10% diluted sample. Moreover, the detected variant frequency was consistent with the expected value (computed from the mutation frequency in each component and the percentage of hepatocarcinoma and sarcoma components at each point) at linear regression analysis, showing that this assay may quantitate the actual allelic frequency of a somatic mutation in a given FFPE sample.

### TM-NGS describes intertumoral and intratumoral molecular heterogeneity

We applied the Ion AmpliSeq Cancer Panel to a series of FFPE samples from 30 additional upper gastrointestinal tract tumors; the series consisted of 5 GC, 5 PDAC, 5 IPMN, 5 AVC, 5 HCC, and 5 ICC. Samples were microdissected to maximize tumor cell percentage. In all samples an adequate library for sequencing was obtained. A mean coverage of 1800x was achieved, with 87.4% target bases covered more than 100x and a mean read length of 78 base pairs.

The spectrum of mutated genes detected in the various tumor types was consistent with the current literature as reviewed in the COSMIC database [18]. *KRAS* mutations were detected in all PDAC and in 3 of 5 AVC and IPMN; *TP53* mutations in 3 of 5 PDAC, 3 of 5 GC and 2 of 5 IPMN, respectively. Other frequently mutated genes were *GNAS* in IPMN (4 of 5 samples), *IDH1* in ICC (2 of 5 samples) and *PIK3CA* in GC and HCC (**Table 2**).

**Table 2:** Mutations detected by amplicon deep sequencing of 46 cancer-related genes hotspots in formalin-fixed, paraffin embedded specimens of 30 upper gastrointestinal tract tumors.

Genes altered in two or more tumor types										
Tumor type *	Neo-plastic cells	*KRAS*	*NRAS*	*BRAF*	*GNAS*	*CTNNB1*	*TP53*	*SMAD4*	*PIK3CA*	*IDH1*
GC1	70%**	G12D (29%)							H1047R (41%)	
GC2	50%**		A66V (29%)							
GC3	75%**						P151S (74%)			
GC4	50%**						M237I (36%)			
GC5	70%**						E339* (33%)		N1044K (28%)	
PDAC1	20%**	G12V (10%)						R135* (12%)	I391M (19%)	
PDAC2	40%**	G12D (21%)			R210H (21%)					R58* (35%)
PDAC3	20%**	G12D (23%)					V272L (31%)			
PDAC4	60%**	G12V (31%)					R282W (51%)	N107Kfs*2 (33%)		L64P (38%)
PDAC5	70%**	G12D (33%)					R196* (19%)			
IPMN1	30%**				R201C (34%)					R132H (19%)
IPMN2	95%**	G12D (27%)			R201C (52%)		I195N (21%)			
IPMN3	50%**	V14I (37%)		K601E (33%)	R201H (38%)					
IPMN4	65%**			T599delinsIP (27%)	R201C (35%)					
IPMN5	80%**	G12D (76%)					R306* (83%)			
AVC1	20%**	G12D (18%)						C499R (13%)		
AVC2	70%**	G12R (84%)			R201C (46%)					
AVC3	50%**					S45F (43%)				
AVC4	40%**							R361H (24%)		
AVC5	60%**	Q61R (31%)					R273C (40%)			
ICC1	95%						R282W (18%)	C115Y (5%)		
ICC2	90%**									R132G (36%)
ICC3	85%**									R132C (24%)
ICC4	80%**		Q61R (25%)	Q461* (9%)						
ICC5	95%**						V274F (72%)			
HCC1	90%					I35S (35%)				
HCC2	90%					S33C (40%)				
HCC3	60%				R201H (13%)					
HCC4	90%					G34V (43%)				
HCC5	40%**								C420R (43%)	

Genes altered in one tumor type							
Tumor type	Neo-plastic cells	*FBXW7*	*STK11*	*ERBB2*	*ATM*	*KDR*	*NOTCH1*
GC1	70%**						
GC2	50%**	R479Q (27%)	F354L# (48%)				
GC3	75%**						V1577L (11%)
GC4	50%**						
GC5	70%**						
PDAC1	20%**						
PDAC2	40%**					Q472H (7%)	
PDAC3	20%**						
PDAC4	60%**						
PDAC5	70%**						
IPMN1	30%**						
IPMN2	95%**				R337C (6%)		
IPMN3	50%**						
IPMN4	65%**						
IPMN5	80%**						
AVC1	20%**						
AVC2	70%**						
AVC3	50%**			V777L (43%)			
AVC4	40%**						
AVC5	60%**						
ICC1	95%						
ICC2	90%**						
ICC3	85%**						
ICC4	80%**						
ICC5	95%**						
HCC1	90%						
HCC2	90%						
HCC3	60%						
HCC4	90%						
HCC5	40%**						

*GC = gastric adenocarcinoma, PDAC = pancreatic ductal adenocarcinoma, IPMN = intraductal papillary mucinous neoplasm, AVC = Ampulla of Vater carcinoma, ICC = Intrahepatic cholangiocarcinoma, HCC = hepatocellular carcinoma.

** Neoplastic cell content refers to the sample after microdissection.

# Germline pathological variant (Peutz-Jeghers syndrome).

Twenty tumors (57%) showed multiple gene alterations with PDAC and IPMN displaying up to four concurring different alterations. In seven cases (20%), significant differences were observed in the frequencies of alterations affecting distinct genes, suggesting the presence of intra-tumor molecular heterogeneity. For example, case ICC4 had a 25% frequency Q61R mutation in the *NRAS* gene coexisting with a 9% of *BRAF* Q461*, while ICC1 showed a 18% frequency R282W *TP53* mutation coexisting with a 5% of *SMAD4* C115Y.

### Orthogonal validation of intratumor molecular heterogeneity detected at TM-NGS by immunolabelling for p53 and Smad4

**Figure 3:**
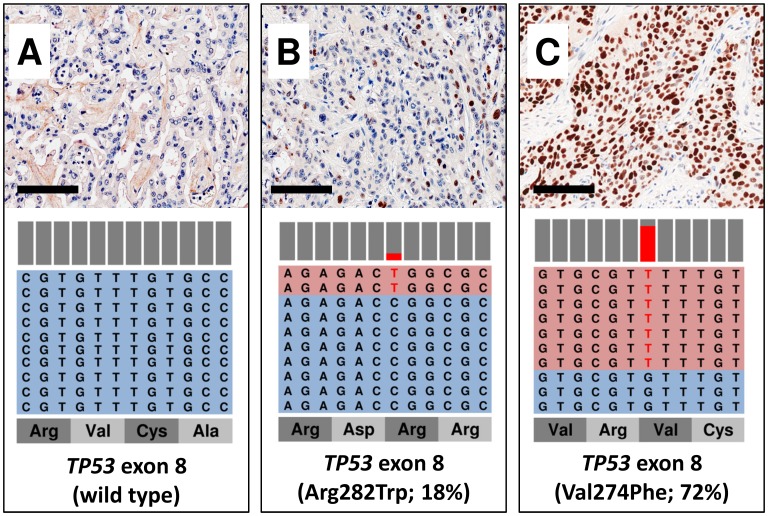
The allelic frequency of mutation in *TP53* gene corresponds to the proportion of p53 immunostained cells. Light blue sequence boxes indicate wild-type amplicons, light red indicates amplicons bearing a mutation, which is highlighted in red. Bars above amplicons show the relative abundance of wild-type (grey) and mutant (red) nucleotides. A) a case with wild type *TP53* showing no p53 immunostaining; B) a case showing about 20% of immunolabelled cells for p53, consistent with *TP53* mutation frequency of 18%; C) a case with *TP53* mutation frequency of 72%, showing a strong and diffuse p53 immunostaining. For each sample a representative H&E and p53 immunohistochemical image (original magnification x20) and the representation of the reads aligned to the reference genome are presented.

**Figure 4:**
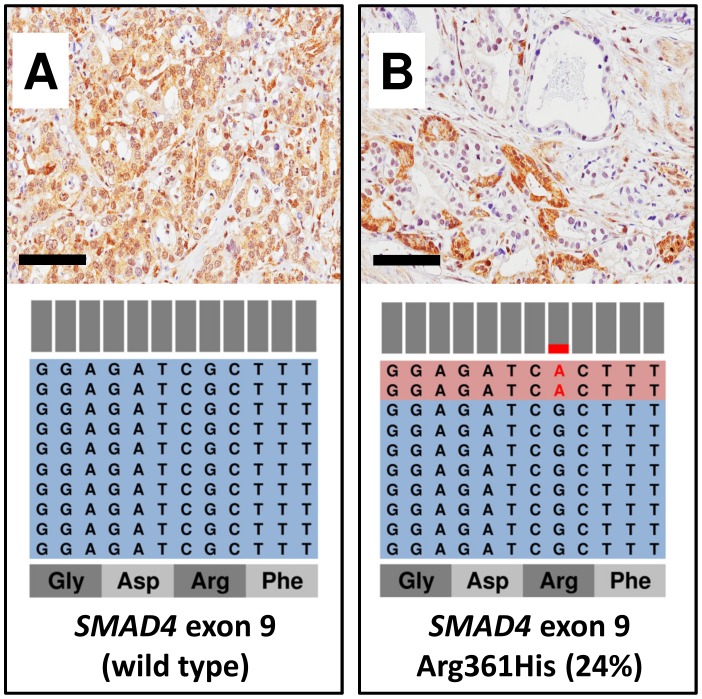
*SMAD4* mutational status corresponds to Smad4 immunohistochemical loss of expression. Light blue sequence boxes indicate wild-type amplicons, light red indicates amplicons bearing a mutation, which is highlighted in red. Bars above amplicons show the relative abundance of wild-type (grey) and mutant (red) nucleotides. A) A case with wild type *SMAD4* showing uniform Smad4 staining. B) A case with *SMAD4* mutation allelic frequency of 24% shows a heterogeneous pattern of immunostaining with alternating positive and negative areas. For each sample a representative H&E and Smad4 immunohistochemical image (original magnification x20) and the representation of the reads aligned to the reference genome are presented.

To validate the relationship between mutation frequency and intratumor heterogeneity, we performed IHC analysis for p53 in ICC and for Smad4 in AVC samples. Case ICC5 (72% mutation frequency) showed a strong and diffuse immunostaining, whereas ICC1 (18% mutation frequency) showed a heterogeneous pattern with sparse or clustered positive cells, roughly accounting for 15% of immunolabelled cancer cells surrounded by regions of negative staining (**Figure 3**). As for Smad4 immunohistochemical analysis in AVC, the tumor sample AVC4 bearing a R361H mutation with 24% frequency displayed a mixture of negative and positive regions, the latter accounting for about 15–20% of cancer cells, while non-mutated samples had a homogeneously positive immunohistochemical pattern (**Figure 4**).

## Discussion

The results of our study may be summarized as follows: TM-NGS can be applied on DNA from routinely prepared paraffin tissues; the data produced are quantitative and thus permit the description of the molecular subclonal composition of a tumor.

The introduction of targeted drugs is changing the profile of information needed to plan a therapeutical approach that entails multiple lines of intervention [19]–[22]. In this scenario, the histopathological diagnosis based on morphological classifications is no longer sufficient, and will need to be complemented by a comprehensive description of the specific molecular alterations and clonal heterogeneity of the tumor [23]–[25]. Proof of concept reports have already shown the potential application of NGS techniques using DNA from FFPE tissues [9], [26]–[29]. However, its introduction in the clinical routine still needs validation of each step leading from the sample to results as well as the design of appropriate panels to specifically interrogate multiple tumor categories.

The present study was therefore designed to evaluate the practicability of TM-NGS in detecting heterogeneity among diverse tumor types of the upper gastrointestinal system. In particular, three issues were addressed: i) to compare the performance of TM-NGS on FFPE-derived partially degraded DNA with that on high molecular weight DNA from fresh-frozen tissues; ii) to assess the mutation detection limit of TM-NGS on both fresh-frozen and FFPE derived DNA; iii) to assess TM-NGS ability in detecting inter-tumor and intra-tumor heterogeneity across upper gastrointestinal tract neoplasms.

We used a commercially available multigene panel that simultaneously investigates the status of mutational hotspots of 46 genes, including oncogenes with available (*EGFR* and *BRAF*) and upcoming (*MET* and *PIK3CA*) targeted therapies, or known to decrease the efficacy of specific personalized therapies (*KRAS*, *NRAS*, *HRAS*).

Mutation detection by TM-NGS was as efficient with the partially degraded DNA from FFPE as it was with high molecular weight DNA from fresh-frozen samples, as shown by the similar coverage and allelic frequency of mutations obtained on matched samples of five SPT. The sensitivity of the assay was assessed by dilution curves, demonstrating that TM-NGS can detect mutated DNA accounting for 2% of the cells in FFPE samples, reaching an even lower detection limit (1%) in fresh-frozen cells/tissues.

While analyzing 35 samples from 7 different tumor types, seven cases with multiple mutations showed significant differences in the frequencies of alterations affecting distinct genes, while in ten cases the allelic frequency of mutations was not consistent with neoplastic cells percentage; this suggested the presence of intra-tumor molecular heterogeneity. Confirmation that TM-NGS quantifies the alleles affected, permitting the description of cancer subclonal composition was obtained by immunohistochemistry: this showed that p53 accumulation or Smad4 loss were seen in a proportion of cells comparable to that indicated by the allelic frequency of the mutation in the corresponding gene.

The prevalence and type of mutations detected are comparable to those expected in the diverse tumor types considered herein, as reported by the curated COSMIC database [18]: the *CTNNB1* gene was always mutated in SPT and in 3 of 5 HCC [17], the R132 hotspot in *IDH1* gene was identified for ICC [30], [31] and *GNAS* R201 for IPMN, *KRAS* was the most frequently mutated gene in pancreatic cancers while *TP53* was frequently mutated in both pancreatic and gastric cancers [15], [32], [33]. Other frequently involved genes included *PIK3CA* and *SMAD4*.

All the 35 tumor samples in our representative series of upper gastrointestinal system cancers were characterized by at least one single specific molecular alteration among the 46 genes analyzed, some of which also represent a potential therapeutic target. Two or more mutations were found in 20/35 (57%) cases. Moreover, several genes were altered in more than one tumor type, suggesting the possibility of a molecular subclassification of tumors that crosses the borders of histology and puts the focus on molecular and potentially actionable alterations [34]. While these commonly altered genes could be detected by the commercial assay used in the present work, additional cross-border molecular alterations or mutations that remain confined to a specific tumor class are being reported [26], [27]. For this reason, the design of specialized and optimized multigene panels will be the next mandatory step. Indeed, a European consortium of research centers has already developed a TM-NGS panel specifically tailored to target colon and lung cancer [14].

In conclusion, our study demonstrates the ability of TM-NGS to detect and quantitate multiple gene alterations, thus moving a further step towards a next-generation histopathologic diagnosis that integrates morphologic, immunophenotypic, and mutational analysis of multiple genes using routinely processed tissues. Morphology and immunohistochemistry will provide diagnosis and drive the choice of areas to be microdissected for multiplex deep sequencing, while aiding the interpretation of sequencing data in light of intratumor heterogeneity. The role of the pathologist will be also critical to ensure the appropriate and ample sampling of the tumor to guarantee a complete and combined histopathologic molecular diagnosis.

Finally, next generation targeted sequencing on paraffin tissue is much less expensive than the sum of many single conventional analyses, while having equal or even higher sensitivity [35]–[37]. This renders clinical application feasible and paves the way to a significant curtail of the economic burden of National Health Services.

## Supporting Information

Table S1 **Clinicopathological characteristics of the series.**
(DOC)

Table S2 **NCBI RefSeq ID of mRNA transcript used for annotation of genetic variants.**
(DOC)
