# Supplementary material for: Reporting Tumor Molecular Heterogeneity in Histopathological Diagnosis
Source: PLoS One. 2014 Aug 15;9(8):e104979. doi: 10.1371/journal.pone.0104979 (PMC4134249; doi:10.1371/journal.pone.0104979)
Supplement: Table S1 — Clinicopathological characteristics of the series. (DOC) [file pone.0104979.s001.doc]

**Table S1.** Clinicopathological characteristics of the series

| **CASE** | **Sex** | **Age** | **Location** | **Diagnosis** | **Size (cm)** | **TNM** |
| --- | --- | --- | --- | --- | --- | --- |
| GC1 | F | 66 | Antrum | Gastric adenocarcinoma (intestinal type) | 8.0 | T2n0m0 |
| GC2 | M | 60 | Antrum | Gastric adenocarcinoma (intestinal type) | 10.0 | T2n0m0 |
| GC3 | M | 47 | Antrum | Gastric adenocarcinoma (intestinal type) | 5.0 | T2n0m0 |
| GC4 | F | 83 | Antrum | Gastric adenocarcinoma (intestinal type) | 3.0 | T2n0m0 |
| GC5 | M | 70 | Fundus | Gastric adenocarcinoma (intestinal type) | 4.0 | T4n1m0 |
| SPT1 | F | 41 | Pancreas tail | Solid pseudopapillary tumor | 12 | - |
| SPT2 | F | 44 | Pancreas tail | Solid pseudopapillary tumor | 4.5 | - |
| SPT3 | F | 24 | Pancreas tail | Solid pseudopapillary tumor | 3.0 | - |
| SPT4 | F | 12 | Pancreas tail | Solid pseudopapillary tumor | 14 | - |
| SPT5 | F | 38 | Pancreas body | Solid pseudopapillary tumor | 5.0 | - |
| AVC1 | F | 27 | Vater’s ampulla | Ampulla of Vater carcinoma | 1.5 | T2n0m0 |
| AVC2 | F | 65 | Vater’s ampulla | Ampulla of Vater carcinoma | 1.5 | T2n0m0 |
| AVC3 | F | 72 | Vater’s ampulla | Ampulla of Vater carcinoma | 3.0 | T2n0m0 |
| AVC4 | M | 68 | Vater’s ampulla | Ampulla of Vater carcinoma | 2.0 | T2n0m0 |
| AVC5 | M | 64 | Vater’s ampulla | Ampulla of Vater carcinoma | 7.0 | T3n1m0 |
| IPMN1 | F | 60 | Pancreas, whole | Intraductal papillary mucinous neoplasm | 1.5 | - |
| IPMN2 | M | 78 | Pancreas head | Intraductal papillary mucinous neoplasm | 1.5 | Tisn0m0 |
| IPMN3 | F | 71 | Pancreas head | Intraductal papillary mucinous neoplasm | 5.0 | T2n1m0 |
| IPMN4 | M | 65 | Pancreas head | Intraductal papillary mucinous neoplasm | 4.0 | T3n1m0 |
| IPMN5 | F | 80 | Pancreas, whole | Intraductal papillary mucinous neoplasm | 3.5 | T2n1m0 |
| PDAC1 | F | 67 | Pancreas head | Pancreatic ductal adenocarcinoma | 3.5 | T3n1m0 |
| PDAC2 | F | 60 | Pancreas body | Pancreatic ductal adenocarcinoma | 4.5 | T3n1m0 |
| PDAC3 | M | 65 | Pancreas head | Pancreatic ductal adenocarcinoma | 3.5 | T4n1m0 |
| PDAC4 | F | 66 | Pancreas head | Pancreatic ductal adenocarcinoma | 3.0 | T3n1m0 |
| PDAC5 | M | 68 | Pancreas head | Pancreatic ductal adenocarcinoma | 1.2 | T3n0m0 |
| ICC1 | F | 53 | Liver | Intrahepatic Cholangiocarcinoma | 7.6 | T2n0m0 |
| ICC2 | M | 62 | Liver | Intrahepatic Cholangiocarcinoma | 7.0 | T2n0m0 |
| ICC3 | M | 51 | Liver | Intrahepatic Cholangiocarcinoma | 16 | T2n0m0 |
| ICC4 | M | 54 | Liver | Intrahepatic Cholangiocarcinoma | 2.7 | T2n0m0 |
| ICC5 | M | 64 | Liver | Intrahepatic Cholangiocarcinoma | 5.0 | T1n0m0 |
| HCC1 | M | 43 | Liver | Hepatocellular carcinoma | 17.0 | T3an0m0 |
| HCC2 | M | 65 | Liver | Hepatocellular carcinoma | 4.0 | T3bn0m0 |
| HCC3 | M | 74 | Liver | Hepatocellular carcinoma | 5.0 | T1n0m0 |
| HCC4 | M | 60 | Liver | Hepatocellular carcinoma | 4.0 | T1n0m0 |
| HCC5 | M | 65 | Liver | Hepatocellular carcinoma | 8.0 | T2n0m0 |
