# Supplementary material for: Reporting Tumor Molecular Heterogeneity in Histopathological Diagnosis
Source: PLoS One. 2014 Aug 15;9(8):e104979. doi: 10.1371/journal.pone.0104979 (PMC4134249; doi:10.1371/journal.pone.0104979)
Supplement: Table S2 — NCBI RefSeq ID of mRNA transcript used for annotation of genetic variants. (DOC) [file pone.0104979.s002.doc]

**Table S2.** NCBI RefSeq ID of mRNA transcript used for annotation of genetic variants

| **Gene (HGNC ID)** | **NCBI RefSeq ID** | **Gene (HGNC ID)** | **NCBI RefSeq ID** |
| --- | --- | --- | --- |
| *APC* | NM_000038 | *KDR/VEGFR2* | NM_002253 |
| *ABL1* | NM_007313 | *KIT* | NM_000222 |
| *AKT1* | NM_001014432 | *KRAS* | NM_004985 |
| *ALK* | NM_004304 | *MET* | NM_000245 |
| *ATM* | NM_000051 | *MLH1* | NM_000249 |
| *BRAF* | NM_004333 | *MPL* | NM_005373 |
| *CDH1* | NM_004360 | *NOTCH1* | NM_017617 |
| *CDKN2A* | NM_000077 | *NPM1* | NM_002520 |
| *CSF1R* | NM_005211 | *NRAS* | NM_002524 |
| *CTNNB1* | NM_001904 | *PDGFRA* | NM_006206 |
| *EGFR* | NM_005228 | *PIK3CA* | NM_006218 |
| *ERBB2* | NM_004448 | *PTEN* | NM_000314 |
| *ERBB4* | NM_005235 | *PTPN11* | NM_002834 |
| *FBXW7* | NM_033632 | *RB1* | NM_000321 |
| *FGFR1* | NM_023110 | *RET* | NM_020975 |
| *FGFR2* | NM_000141 | *SMAD4* | NM_005359 |
| *FGFR3* | NM_000142 | *SMARCB1* | NM_003073 |
| *FLT3* | NM_004119 | *SMO* | NM_005631 |
| *HNF1A* | NM_000545 | *SRC* | NM_005417 |
| *HRAS* | NM_005343 | *STK11* | NM_000455 |
| *IDH1* | NM_005896 | *TP53* | NM_000546 |
| *JAK2* | NM_004972 | *VHL* | NM_000551 |
| *JAK3* | NM_000215 |  |  |
